# Spatial interaction mapping of PD-1/PD-L1 in head and neck cancer reveals the role of macrophage-tumour barriers associated with immunotherapy response

**DOI:** 10.1186/s12967-025-06186-y

**Published:** 2025-02-12

**Authors:** Vahid Yaghoubi Naei, Rafael Tubelleza, James Monkman, Habib Sadeghirad, Meg L. Donovan, Tony Blick, Agata Wicher, Sara Bodbin, Amelie Viratham, Robert Stad, Subham Basu, Caroline Cooper, Catherine Barnett, Ken O’Byrne, Rahul Ladwa, Majid Ebrahimi Warkiani, Brett G. M. Hughes, Arutha Kulasinghe

**Affiliations:** 1 School of Biomedical Engineering, University of Technology Sydney, Sydney, NSW Australia; 2 Frazer Institute, Faculty of Medicine, The University of Queensland, Brisbane, QLD Australia; 3 Queensland Spatial Biology Centre, Wesley Research Institute, The Wesley Hospital, Brisbane, Australia; 4 Navinci Diagnostics, Uppsala, Sweden; 5 Akoya Biosciences, Menlo Park, CA USA; 6 The Princess Alexandra Hospital, Brisbane, Australia; 7 The Royal Brisbane and Women’s Hospital, Brisbane, Australia; 8 School of Medicine, University of Queensland, Brisbane, Australia

**Keywords:** Head and neck cancer, Proximity ligation assay, Immunotherapy, Macrophages, Cellular interactions, PD-L1 interactions

## Abstract

**Background:**

Mucosal head and neck squamous cell carcinoma (HNSCC) is often diagnosed at an advanced stage, where the prognosis is poor due to the high rates of recurrence and metastasis. With approximately one million new cases projected in 2024, worldwide mortality of HNSCC is estimated to reach 50% of detected cases the same year. Patients with early-stage tumours showed a 50–60% five-year survival rate in the US. Immune checkpoint inhibitors (ICIs) have shown promising results in prolonging survival in a subset of patients with recurrent or metastatic disease. However, challenges remain, particularly the limited efficacy of PD-1/PD-L1 blockade therapies. PD-L1 protein expression has been shown to be limited in its predictive power for ICI therapies. Emerging evidence shows that intricate characterisation of the tumour microenvironment (TME) is fundamental to understand interacting cells. This study aims to bridge the gap in understanding the tumor microenvironment by identifying distinct spatial patterns of PD-1/PD-L1 interactions and their association with immunotherapy responses in head and neck squamous cell carcinoma (HNSCC).

**Methods:**

In this study, we sought to apply a more nuanced approach to understanding cellular interactions by mapping PD-1/PD-L1 interactions across whole-slide HNSCC tissue samples collected prior to ICI therapy. We used a combination of spatial proteomics (Akoya Biosciences) and an in situ proximity ligation assay (isPLA, Navinci Diagnostics) to visualise PD-1/PD-L1 interactions across cell types and cellular neighbourhoods within the tumour TME.

**Results:**

Our findings indicate the existence of isPLA^+^ PD-1/PD-L1 interactions between macrophages/CD3 T cell-enriched neighbourhoods and tumour cells at the tumour-stroma boundaries in ICI-resistant tumours. The presence of these dense macrophage-tumour layers, which are either absent or dispersed in responders, indicates a barrier that may restrict immune cell infiltration and promote immune escape mechanisms. In contrast, responders had abundant B and T cell aggregates, predominantly around the tumour edges linked to enhanced immune responses to ICI therapy and better clinical outcomes.

**Conclusion:**

This study highlights the utility of isPLA in detecting distinct tumour-immune interactions within the TME, offering new cellular interaction metrics for stratifying and optimising immunotherapy strategies.

**Supplementary Information:**

The online version contains supplementary material available at 10.1186/s12967-025-06186-y.

## Introduction

Head and neck squamous cell carcinoma (HNSCC) arises from the mucosal linings of the oral cavity, sinonasal cavity, pharynx and larynx. It ranks as the eighth most prevalent cancer worldwide with more than 800,000 new cases and resulting in about 450,000 deaths yearly [1] The main risk factors for HNSCC include tobacco, excessive alcohol consumption and human papillomavirus (HPV) infection [2, 3]. Utilising a combination of surgery, radiotherapy and systemic therapies, patients have a 5-year survival rate of 50-70% [4, 5]. Recent studies have shown that immune checkpoint inhibition offers a promising new therapeutic option for advanced/recurrent HNSCC [6, 7].

Immune checkpoint inhibitors (ICI) regulate the innate immune response. Tumour-cell expression of programmed cell death-ligand 1 (PD-L1) interacts with its receptor (PD-1) on tumour-specific T lymphocytes, limiting their anti-tumour efficacy [8]. Novel PD-1/PD-L1 inhibitors prevent this interaction, allowing for increased lymphocyte proliferation and effector anti-tumour function [9]. Anti-PD-1 and anti-PD-L1 antibodies demonstrated encouraging effects in a variety of cancer types [10, 11]. The US Food and Drug Administration (FDA) has approved nivolumab and pembrolizumab as therapies for patients with recurrent or metastatic HNSCC [12, 13]. However, ICI benefits less than 30% of patients [14], highlighting the need to better predict those patients who will benefit from ICI therapy. Eligibility for ICI therapy is often determined by PD-L1 protein expression, evaluated by two scoring systems: the Combined Positive Score (CPS) and the Tumour Proportion Score (TPS) [15]. The CPS is computed by dividing the overall number of PD-L1-stained cells (comprising tumour cells, lymphocytes, and macrophages) by the total number of viable tumour cells and multiplying the result by 100 [16]. Conversely, TPS solely evaluates PD-L1 expression in tumour cells as a proportion of the overall tumour cell population [17, 18]. CPS encompasses both tumour and immune cells, potentially offering a more accurate representation of the immunological microenvironment; nonetheless, both scoring systems have limitations. CPS can overstate PD-L1 expression by incorporating immune cells that do not directly influence therapeutic response, whereas TPS may overlook significant immunological interactions [19]. Despite these constraints, both scores are utilised to guide ICI therapy in various cancers, although CPS is currently used in HNSCC. Targeting the PD-1 axis using ICI has become a cornerstone for treating recurrent and metastatic HNSCC [6]. Emerging approaches, such as in situ proximity ligation assays, are providing deeper insights into the spatial dynamics of PD-1/PD-L1 interactions within the TME [6]. Additionally, the potential benefits of neoadjuvant PD-1/PD-L1 inhibitors in resectable HNSCC have been explored, showing promising results in improving outcomes for patients undergoing surgery, showing the need for continued exploration of the tumor microenvironment to advance therapeutic strategies and improve patient outcomes [20, 21].

Spatial proteomics has been named the Nature “Method of the Year” in 2024, highlighting the technological advancements to profile the TME. In this study, we utilised the in situ Proximity Ligation Assay to spatially map PD-1/PD-L1 interactions in a cohort of HNSCC samples prior to ICI therapy. Cell types were mapped and their corresponding neighbourhoods were identified before being integrated with spatial interaction metrics for PD-1/PD-L1 [22]. This cutting-edge approach provided detailed insights into the TME which enabled us to compare responders and non-responders to ICI therapy with recurrent or metastatic (R/M) HNSCC to identify spatial patterns associated with therapeutic response [23, 24].

## Materials and methods

### Patients and samples

This retrospective cross-sectional study used formalin-fixed, paraffin-embedded (FFPE) tissues collected via biopsy/surgical resection from a cohort of *n* = 35 Recurrent/Metastatic HNSCC patients from the Princess Alexandra Hospital and Royal Brisbane & Women’s Hospital, prior to being treated with anti-PD-1 immunotherapy (Table 1). This study has Human Research Ethics (HREC) approval from the Royal Brisbane and Women’s Hospital (RBWH) (LNR/2020/QRBW/66744) and The University of Queensland Human Research Ethics ratification. Patient treatment response was determined using the RECIST 1.1 criteria [25]. According to this, patients are classified as complete response (CR), partial response (PR), stable disease (SD), and progressive disease (PD).

**Table 1:** Patient’s clinical information

Gender	
Male	28 (80%)
Female	7 (20%)
Age	
Median (Min, Max)	67 (29,81)
Response group	
Complete Response (CR)	4 (11%)
Partial Response (PR)	6 (17%)
Stable disease (SD)	7 (20%)
Progressive Disease (PD)	18 (52%)
Survival status	
Alive	1 (3%)
Deceased	27 (77%)
Unknown	7 (20%)
Treatment	
Nivolumab	29 (82%)
Pembrolizumab	6 (18%)
P16 status	
Positive Negative NA (oral cavity)	6 (17%) 2 (6%) 6 (17%)
Unknown	21 (60%)
ECOG performance	
0 1 Unknown	4 (11%) 11 (31%) 20 (57%)
Smoking status	
Current/former smokers	12 (34%)
Non-smokers	2 (6%)
Unknown	21 (60%)

### Tissue preparation

Adjacent serial sections were used for the staining and analysis process, followed by antigen retrieval. The selected serial sections were baked, dewaxed and rehydrated through several rounds of xylene, alcohol, and distilled water washes as described previously [26]. Next, antigen retrieval was performed by heating the samples at 120 °C in citrate buffer (pH 6) using a pressure cooker for 15 min, followed by immersion in Tris-buffered saline with Tween 20 (TBS-T) detergent.

### PhenoCycler fusion high plex staining and imaging

**Fig. 1:**
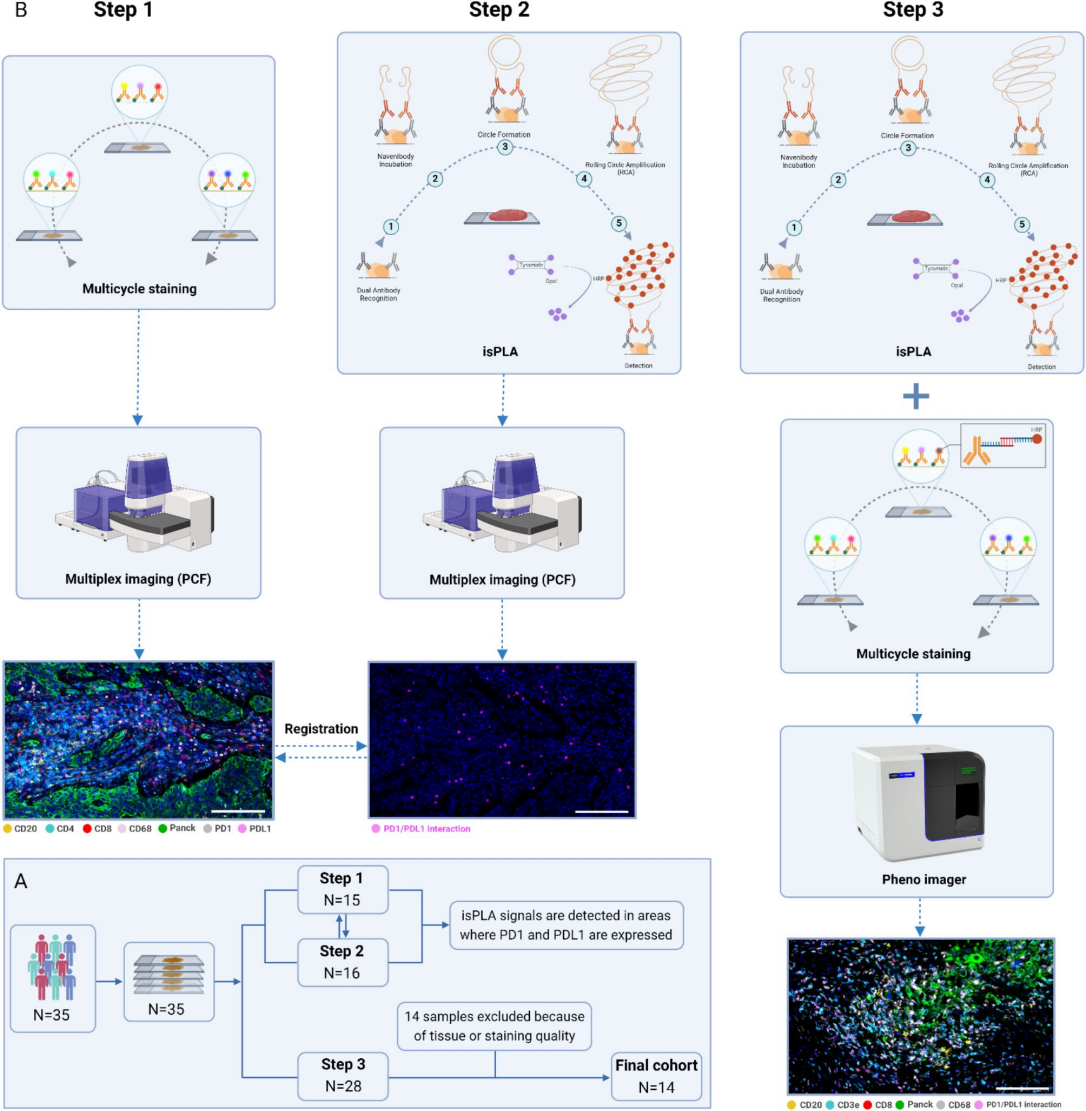
Summary of workflow. **A**) A cohort of 35 HNSCC patients was selected, with serial sections per patient. In step 1, 15 serial sections were processed using a 53-plex panel of antibodies with PCF to visualize PD-1 and PD-L1 expression as well as the distribution of various cell types across tissues (**B**, left panel). In step 2, serial sections from 16 patients were stained using isPLA kit and scanned by PCF to detect PD-1/PD-L1 interactions (**B**, middle panel). the resulting images from steps 1 and 2 were registered to ensure isPLA signals corresponded to regions with PD-1 and PD-L1 expression. In step 3, an exploratory cohort of 28 serial sections were stained with a 6-plex panel and imaged using the PhenoImager system to identify interacting cells through PD-1/PD-L1 across whole tissues and per different response groups to immunotherapy (**B**, right panel)

Using a 53 plex panel of antibodies, we stained 15 serial sections via a sequential round of staining, each targeting a specific set of protein markers [26]. Upon each cycle, the slides were imaged with three markers, and fluorophores were removed to allow for the next rounds. After all 53 markers were imaged, the resulting qptiff file was exported for image analysis (Fig. 1).

### Control samples: tonsil tissues for isPLA validation

**Fig. 2:**
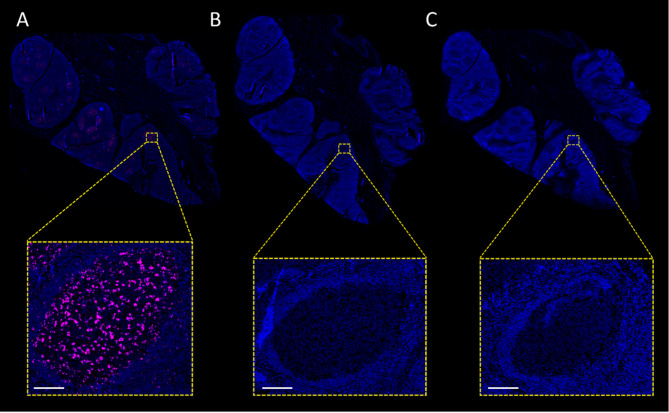
Positive and negative controls. (**A**) Strong interaction PLA signals as a positive control for the PD-1/PD-L1 interactions. This image shows the germinal centers where it is expected to see PD-1/PD-L1 interactions between B and T cells. (**B**) Omitting the PD-L1 antibody and performing isPLA using the anti-PD1 antibody and a rabbit isotype control reveals no background or non-specific binding in these negative controls. (**C**) Staining with anti-PD-L1 and mouse isotype control with the PD-1 antibody omitted shows no background or non-specific binding in these negative controls. Scale = 100 μm

The assay’s specificity was confirmed on germinal centers within tonsil tissue and through the inclusion of isotype control antibodies as negative controls, which resulted in minimal background noise (Fig. 2A-C).

### In situ proximity ligation assay (isPLA) and protein spatial profiling (PSP)

Following the manufacturer’s instructions, 28 whole HNSCC tissue samples were assessed for PD-1/PD-L1 interactions using the Naveni^®^ PD-1/PD-L1 Atto647N kit. To begin the process, slides were initially blocked for 60 min at 37 °C with a Naveni blocking solution. They were then incubated with PD-1 (Cell Signaling Technology, 1:40) and PD-L1 (Abcam, 1:40) primary antibodies for one hour at 37 °C. Secondary antibodies conjugated to oligos, known as Navenibodies, were then added for 60 min at 37 °C, enabling circle formation (reaction 1) and rolling circle amplification (reaction 2). The slides were subsequently treated with Naveni reaction solutions: reaction 1 at 37 °C for 30 min, followed by reaction 2 at 35 °C for 60 min. HRP was applied to the slides for 30 min at room temperature, and the interaction product was identified using Opal-TSA chemistry.

### Opal multiplex imaging

The PhenoCode Signature Immune Profile Human Protein Panel was employed, utilizing Tyramide Signal Amplification (TSA). The application of Opal dyes in the TSA method enhances protein detection by using HRP to convert Tyramide into reactive radicals at specific antigen sites [27, 28].

Slides were stained using the Leica BOND^®^ RX autostainer (Leica, UK) with primary antibodies for CD3e (AKYP0125, 1:12000), CD8 (AKYP0028, 1:2000), CD20 (AKYP0049, 1:800), CD68 (AKYP0050, 1:8000) and Pan-CK (AKYP0053, 1:4000), according to the manufacturer’s instructions. In brief, after dewaxing, epitope retrieval, and blocking, tissue sections were stained with the PhenoCode Signature panel antibody cocktail. Antibodies are conjugated to unique oligo barcodes and applied to tissue in a single cocktail incubation step. Single antibodies were revealed one at a time, beginning with the hybridisation of a complementary oligo conjugated to HRP. Signal amplification was performed using Opal chemistry. The detection process was repeated for each antibody until all Opal dyes had been deposited onto the tissue. Finally, sections were counterstained with DAPI and mounted using Fluoromount-G (Invitrogen ProLong Diamond Antifade Mountant, Thermo Fisher Scientific, USA). Imaging and spectral unmixing were performed using the PhenoImager^®^ HT 2.0 (Akoya Biosciences, USA) and Inform software.

### Image analysis

Images were loaded into QuPath [29] and then segmented on the DAPI2 channel using the Cellpose plugin [30]. The ‘cyto2’ pre-trained model was used with a cell expansion of 3 µm and a cell constraint scale of 1.5. The accuracy of cell segmentation was visually checked. Tissue sections with substandard quality, characterised by fragmentation, folding, or necrosis, and non-specific fluorescence, were excluded. A pixel classifier using an artificial neural network (ANN) was trained on the pan-cytokeratin (PanCK) signal to create a tumour/stroma annotation mask to classify PanCK positive pixels as ‘tumour’ regions. An annotation of ‘tumour’ was only assigned to tumour nests larger than 100 µm^2^. Cell metrics like universally unique identification (UUID) codes, median cell expression, nuclear size, and spatial coordinates for each channel were exported for analysis in Python. The cell classifications obtained via unsupervised clustering were imported back into QuPath for cell visualisation and quality control.

### isPLA positivity classification

A QuPath-trained object classifier was employed to classify cells into positive and negative categories based on the intensity of isPLA signals. To establish the classification criteria, the signal intensity was visually inspected across all slides and annotated as positive and negative. For classifier training, the ‘train object classifier’ option was used to select cell features relevant to PD-1/PD-L1 interactions, such as intensity thresholds for each signal. An appropriate threshold was set for all slides to maintain consistency and applied systemically on all slides to label cells as positive or negative.

### Cell classification

Expression matrices and cell metadata were converted to Anndata [31, 32] format for quality control, preprocessing, clustering, and cell phenotyping. Artifactual nuclei were eliminated by setting a minimum median DAPI signal threshold and then excluding nuclei with sizes smaller than 10µm^2^ and larger than 220µm^2^. The expression matrices of all 6 markers underwent an arcsinh transformation with a cofactor of 150. They were then scaled first within columns (markers) and subsequently across rows (cells), following the suggested PhenoCycler Fusion pre-processing methods [26]. The data was merged using the Scanpy implementation of Harmonypy [33]. The principal components (PCs) were subsequently employed to group the data using Phenograph [34], with a value of k = 30 and a Leiden resolution of *r* = 2, chosen after assessing resolutions 1 to 4 for effectiveness in distinguishing cell types. Clustering was conducted using a set of six markers, namely PanCK, CD20, CD3e, CD8, CD68, isPLA and nuclear area (µm²). Canonical cell-type markers were used for cell typing, and Phenograph determined the functional subsets B cells (CD20^+^), pan T-cells (CD3e^+^), cytotoxic T cells (CD8^+^), tumour cells (PanCK^+^), Macrophages (CD68^+^) and PD-1/PD-L1 interactions (isPLA^+^) in a single clustering iteration.

### Cell analysis

Cell frequency was determined by combining the occurrences of each cell category and normalising for the total number of cells in each tissue or region of the tumour/stroma, resulting in cell percentages. The spatial pscore function in Scimap (https://github.com/labsyspharm/scimap) was used for interaction analysis. The analysis was conducted with the method set to “radius” and a radius of 20 μm. The proximity density metric in the spatial pscore function is determined by calculating the ratio of pairwise interactions to the number of cells in each cell-pair per sample, essentially quantifying each sample based on the fraction of a certain cell pair colocalising within a 20 μm radius.

### Cellular neighbourhood analysis

Cellular neighbourhoods (CNs) were characterised as previously described [35]. A window of a cell’s 10 closest neighbours was used to construct a feature matrix, where columns represented the frequency of observing a given cell type in that window. KMeans clustering was performed to identify CNs, with the optimal number of CNs or K clusters assessed over a range of K values from 3 to 15. For each K value, the sum of squared distances of cells to the centroid of their assigned cluster (inertia) was computed and used as a proxy for clustering coherence or ‘tightness’. The elbow point in the relationship between inertia and K was used to choose an optimal K, as this represented a clustering run that minimised both K and inertia. A Python implementation of the Kneedle algorithm [36, 37] was used to systematically identify elbow points (supplementary Fig. S1A, B). Two different CN variations were computed using cell type labels, with and without including the isPLA-positivity metric.

### Spatial context analysis

Spatial contexts (SCs) were identified using SPACEc [38] to represent regions where multiple CNs potentially interface with each other through local processes [39]. SCs were computed by considering a large window size of each cells’100 closest neighbours and computing the frequencies of observing CNs in that window. The minimal combinations of CNs, or SCs, were identified by first sorting the CNs by abundance. If the most abundant CN was composed of more than 90% of the cells in the window, then this was considered a minimal combination. Otherwise, the process was done recursively, adding the next most abundant CN to the combination until it exceeded the threshold of 90%. The SCs were then connected to form a hierarchical SC map for visualisation. Separate SC maps were computed for each individual patient group.

### SC maps

SC maps were visualised using SPACEc. These maps visualise the hierarchical and agglomerative nature of SCs and the various combinations of CNs that compose them in the form of a directed graph, where nodes represent combinations and directed edges represent a more complex combination from which a source combination was used. A separate map was produced for each patient grouping.

### CN interface analysis

The interfaces between 3 CNs was visualised with a barycentric coordinate projection using SPACEc. For this visualisation, the windows were filtered to those that had at least 99.95% of cells being composed of the Tumour, PLA-, Tumour & immune, PLA^+/−^, and/or Tumour and immune, PLA + CNs. Cells within these windows are then placed on the barycentric coordinate system, based on the relative composition of their window. Cells placed on the corners indicate that they come from a window of mainly one of these CNs. Cells placed on the edges indicate that they reside in an interface window or edges between 2 of these CNs. Cells in the middle come from a window where all 3 CNs are intermixed. These cells are coloured by their CNs. A separate projection was produced for each patient group.

## Results

**Fig. 3:**
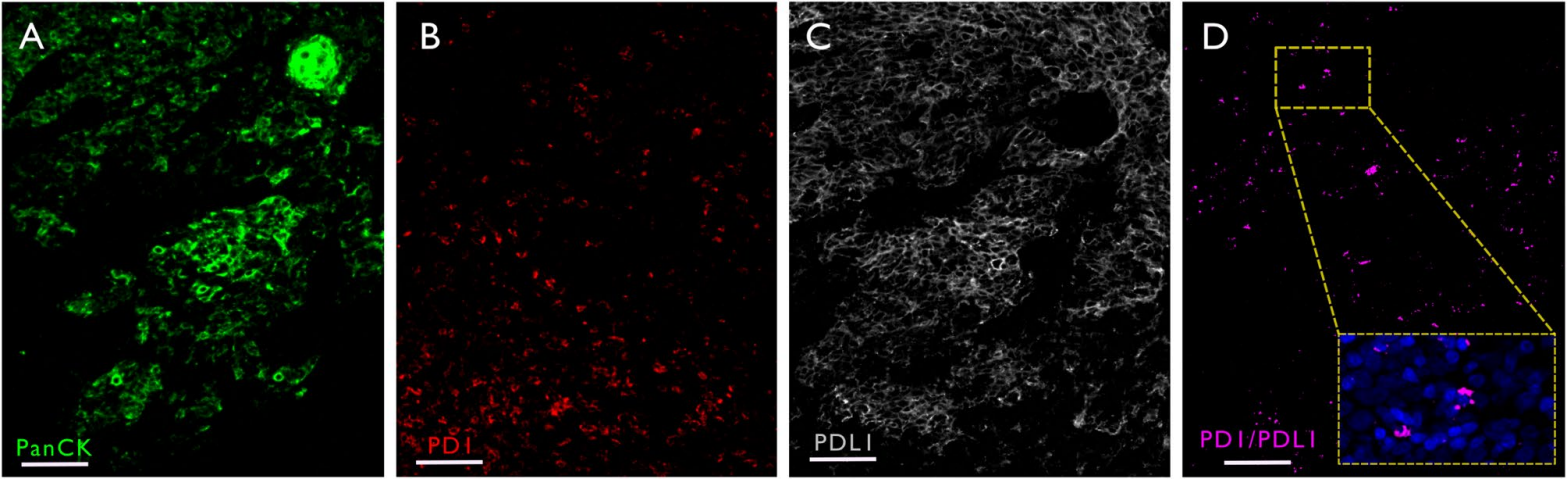
isPLA signals registration and classification. **A**-**C**) Individual IF channels for PanCK, PD-1, and PD-L1 acquired by PCF, compared to **D**) which shows the same region with positive isPLA signals resulting from PD-1/PD-L1 interactions. Scale = 50 μm

The current study used a novel proximity ligation technology to confirm and spatially map PD-1/PD-L1 interactions within a cohort of 35 HNSCC patients. To validate the assay’s specificity, we initially used a high plex staining panel that identified PD-1, PD-L1 and various other immune and non-immune cell markers, enabling us to differentiate cell types across the tissue and estimate the regions with a higher chance of isPLA interactions. The assay was then applied to a serial tissue section, and co-registered images were obtained, revealing highly localised PD-1/PD-L1 interactions occurring within a 40 nm distance between the ligand and the receptor, as shown in Fig. 3D. Due to challenges with image registration, subsequent experiments were performed using Opal multiplex chemistry, which allowed the detection of isPLA interactions alongside key immune markers on the same slide.

To do this, we applied isPLA to 16 sections, PCF to 15 sections and PSP to 28 sections. Specifically, 11 sections were processed using PSP & isPLA, 11 with PSP + PCF, 10 with isPLA & PCF, and 8 samples were processed using all three methods.

### Specificity and accuracy of PD-1/PD-L1 detection

isPLA was applied to detect PD-1 and PD-L1 interactions within the clinical samples using 16 sections of whole tissues, which were processed using the Navini Atto647N kit and scanned using Akoya PhenoCycler Fusion (PCF, Akoya Biosciences). Serial sections of the same tissues were stained using a 53-plex panel of antibodies, including PD-1 and PD-L1, CD4, CD8, CD19, CD21, CD20, CD68, CD163, CD14, CD45, CD15, PanCK and some other markers. This allowed us to identify PD-1 and PD-L1 expression regions across the tissue, estimating the likelihood of detecting isPLA interactions. Staining quality and background signals were visually inspected. Any tissues showing non-specific staining, low signals or background signals were excluded. Images taken from serial sections of the same tissues were subsequently aligned using Warpy in Qupath to confirm that isPLA signals are detected in the same regions as PD-1/PD-L1 expression regions (Fig. 3A, D).

### Multiplex image analysis workflow

Due to the challenges of registering images and combining isPLA and multiplex imaging on the same slide, serial sections of 28 samples were stained for CD8, CD3e, CD68, CD20, PanCk and isPLA. Following cell segmentation, cells were classified into tumours and stroma. Images were visually reviewed to verify tissue quality for analysis, resulting in the exclusion of 14 samples due to tissue fragmentation, out-of-focus regions and staining/tissue quality. Cell phenotyping was carried out via unsupervised clustering based on canonical marker expression. These cell phenotypes were further analysed using spatial approaches to compare to clinical factors.

### Cell phenotyping

The analysis of ~ 10^7^ cells from 14 HNSCC tissue sections resulted in 5 phenotyped clusters according to their predominant canonical marker expression; B cells, CD8^+^ T cells, macrophages, CD3^+^ T cells and tumour cells.

### isPLA signal distribution and intensity analysis based on distance from tumour regions

This analysis focuses on identifying clear patterns of response in pre-defined clinical groups. Stable disease samples present a particular challenge in clinical studies because patients in this category show neither significant tumour shrinkage nor noticeable progression. This ‘in-between’ state makes it difficult to accurately assess the biological effects of ICI when compared to those with progressive disease or patients who demonstrate a clear response, whether partial or complete.

To identify the regions with the highest frequency of PD-1/PD-L1 interactions, we analysed the distribution of distances of PLA signals tumour from the tumour boundary. Signed distances, both inside and outside the edge of the tumour annotation were calculated for four different immune cell types; B cells, CD3^+^ T cells, CD8^+^ T cells and macrophages, for clinical response groups (CR = 2, PD = 6, SD = 2, PR = 4).

**Fig. 4:**
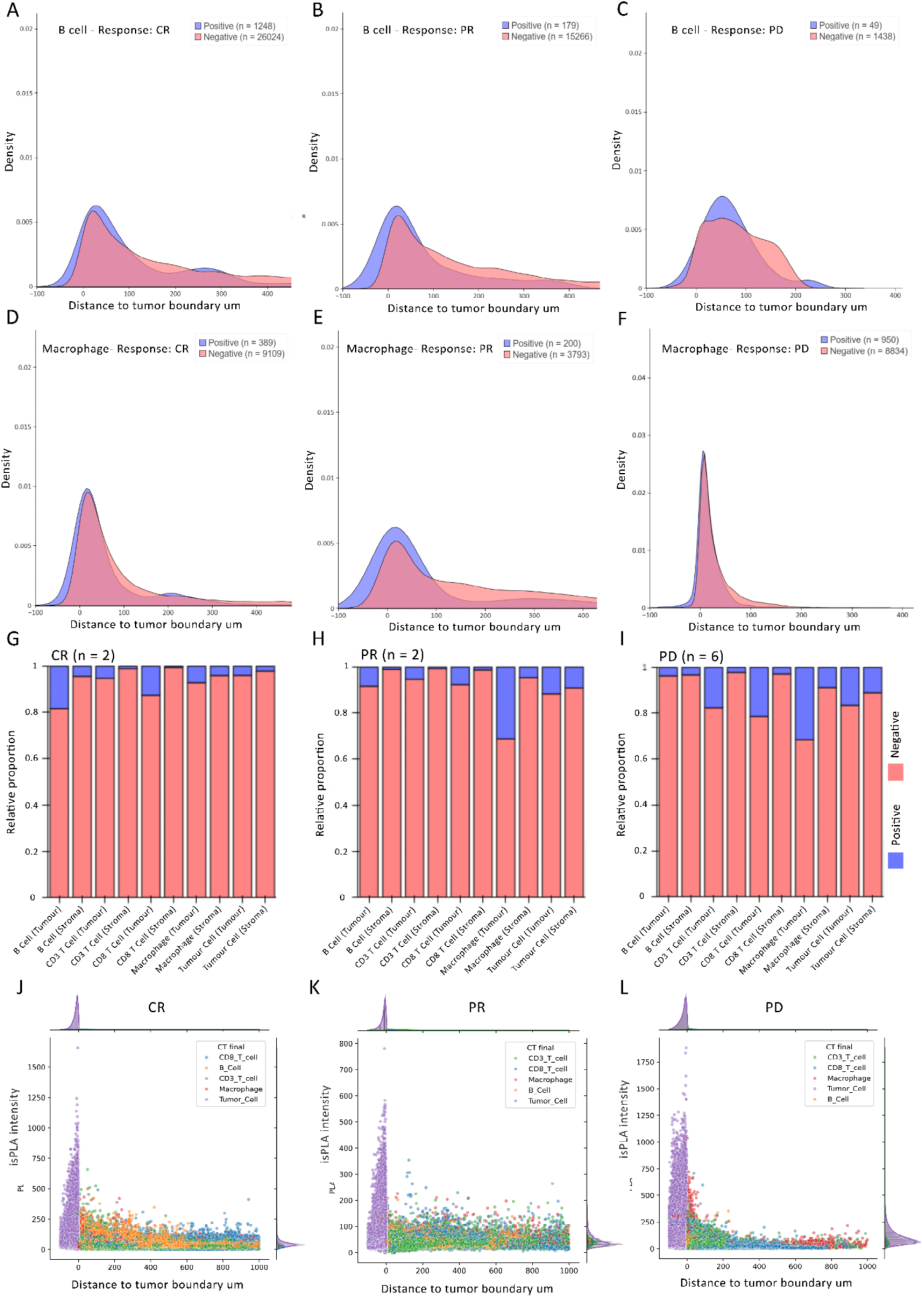
Density plots showing the distance to the tumour boundary, normalized cell count, and isPLA status for B cells (**A**-**C**) and macrophages (**D**-**F**) across response groups. For both B cells and macrophages, more isPLA^+^ cells are located near the tumour boundary in the PD group. Comparison of isPLA^+^ cell types per region shows that tumour-infiltrating B cells are the most frequent interactors with tumour cells via PD-1/PD-L1 in CRs (**G**), while macrophages (**H** & **I**) and CD8 T cells dominate in tumour regions of PDs (**I**). Scatter plots across response groups reveal a higher concentration of B cells with strong isPLA intensity near the tumour boundary in the CR group (**J**) and no specific distribution pattern was observed in the PR group (**K**), while macrophages show a similar pattern in the PD group (**L**)

For each response group, histograms illustrate the relative abundance of isPLA signals as a function of distance from the tumour boundary (Fig. 4A-F). The observed distribution patterns of PD-1/PD-L1 ligation signals (reveal distinct differences in signal density between response groups and cell types), with positive signals being more densely concentrated closer to the tumour boundary in the PD group compared to other response groups, suggesting a potential role of PD-1/PD-L1 interactions in mediating immune responses. In PD patients, most isPLA^+^ B cells are distributed within 300 μm of the tumour boundary, whereas macrophages are more densely clustered within 200 μm of the tumour (Fig. 4C, F). A comparable distribution pattern is observed for CD3 and CD8 T cells (Supplementary Fig. S2). Conversely, a broader distribution of all cell types was found for other response groups, such as PR at the tumour edge, extending beyond 400 μm from the tumour boundary (Fig. 4A, B, D and E).

Integrating distance, response, and isPLA intensity into a scatter plot highlights a trend that isPLA signal intensity is higher within 200 μm of the tumour boundary across all response groups except PR (Fig. 4K). In PD patients, high isPLA signal macrophages and CD3 T cells cluster near the tumour boundary, while CR’s B cells exhibit the highest isPLA signal intensity, in the tumour-stroma interface (Fig. 4J, L).

### isPLA positivity in immune and tumour cells across response groups

The relative proportions of isPLA positivity for each cell type (adjusted to 0 to 1) were compared across response groups in both tumour and stromal regions using multiple Kruskal-Wallis tests. Although no features reached statistical significance after correcting for multiple comparisons, some potential biologically relevant trends were reported. In the CR group, B cells (0.2) and CD8 T cells (0.15) within tumour regions exhibited the highest isPLA positivity, indicating more PD-1/PD-L1 interactions between these cells and tumour cells (Fig. 4G). In the PD group, CD3e cells (0.18), CD8 T cells (0.22), and macrophages (0.35) within tumour regions showed the highest isPLA positivity, suggesting a potentially greater immune-suppressive capacity of the tumour microenvironment in these patients (Fig. 4I). The PR group exhibited similar levels of isPLA positivity in tumour-infiltrating macrophages to the PD group, but markedly lower proportions in CD3e and CD8 T cells (Fig. 4H).

### Cellular neighbourhoods

**Fig. 5:**
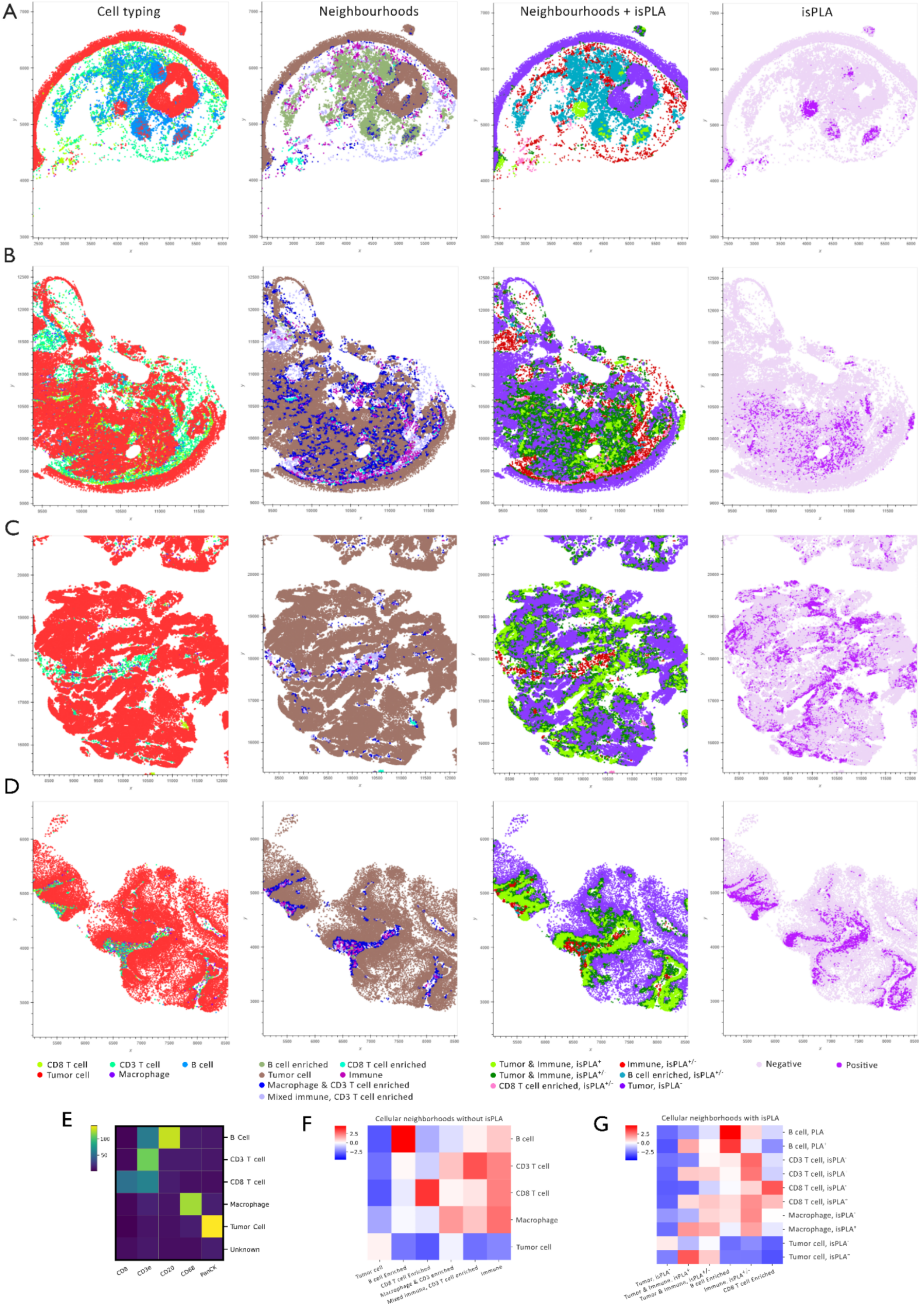
Cell typing and cellular neighbourhoods across response groups (CR (**A**), PR (**B**), SD (**C**), PD (**D**)). Comparison of cellular neighbourhoods with and without including isPLA^+^ clusters showing a different isPLA^+^ neighbourhoods distribution across different response groups. **E**) The cell typing heatmap shows the expression markers’ intensity vs. proposed cell types. **F**, **G**) Cell types were clustered into unsupervised cellular neighbourhoods according to their nearest neighbours. The heatmap shows an abundance of cell types in each neighbourhood with and without including isPLA positivity

Networks of individual cells define the functional features of tissues. Cellular neighbourhoods were characterised by performing unsupervised spatial clustering of cell types by their six nearest neighbours (Fig. 5E-G). Tissue analysis of the relative composition of each neighbourhood revealed that more B cell neighbourhoods are enriched around the tumour boundary in responders (Fig. 5A, second panel), while in non-responders, more dispersed immune neighbourhoods were observed with a higher prevalence of macrophage and CD3 T cell neighbourhoods close to the tumour periphery (Fig. 5D, second panel).

Two distinct CN variants were calculated utilising cell type labels augmented with and without isPLA-positivity. A notable increase in tumour & immune cell isPLA^+^ neighbourhoods and tumour & immune cell isPLA^+/−^ neighbourhoods was observed at the tumour boundary within the PD group (Fig. 5D, third panel). These aggregations created dense layers that were either lacking or minimal in other response groups, such as PR or CR (Fig. 5B, A).

### Spatial contexts (SCs)

Spatial contexts (SCs) refer to areas where distinct local processes occur between neighbouring regions through direct interactions or molecular exchanges. These interactions may lead to specialised biological activities that are unique to these neighbourhoods and are thought to play a crucial role in shaping complex tissue behaviors.

**Fig. 6:**
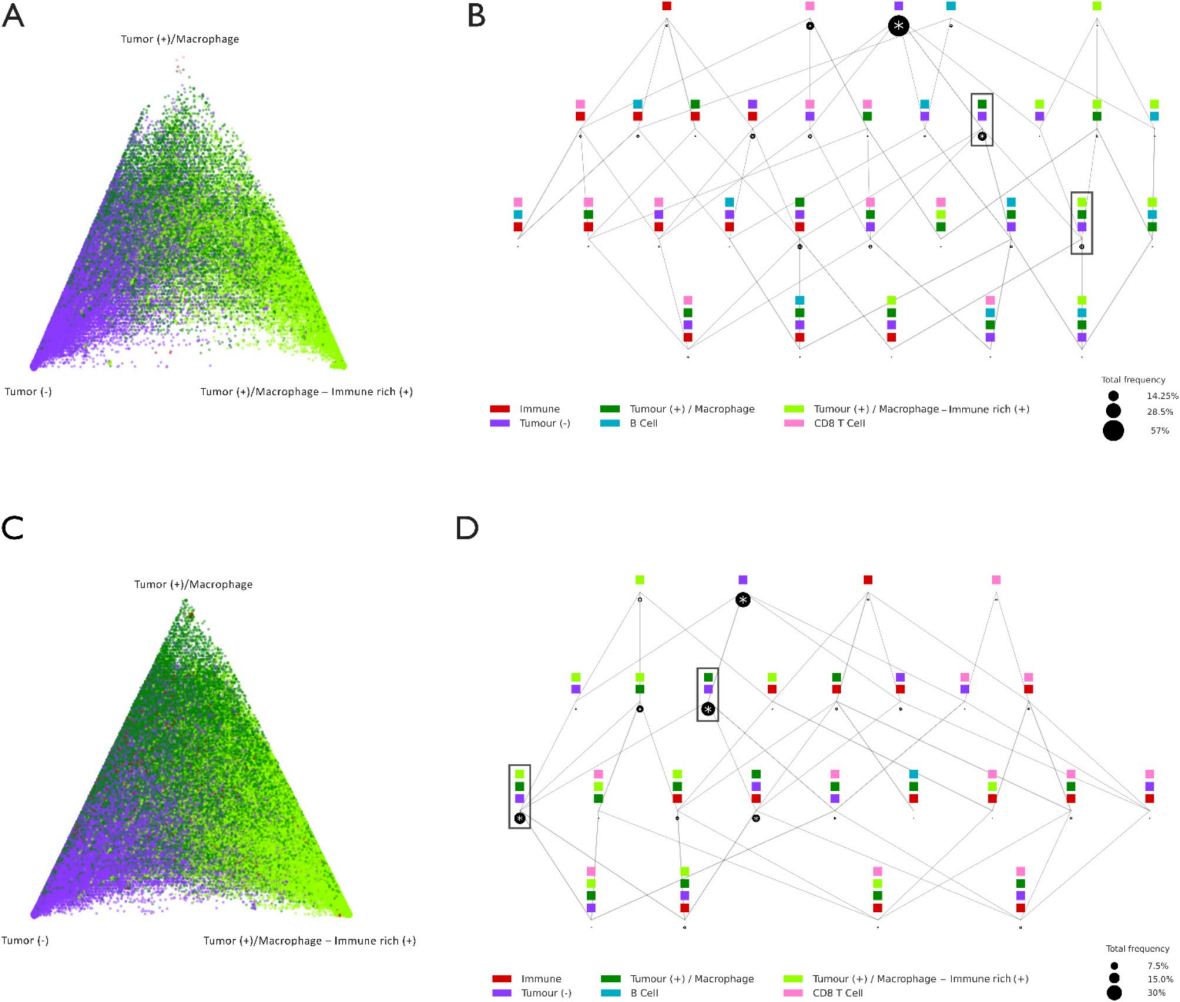
Spatial contexts as sites of functional interactions between CNs colocalization. (**A**) Barycentric coordinate projection of windows of cells assigned to Tumour, isPLA^−^, Tumour & immune, PLA^+/−^, and Tumour and immune, isPLA^+^. Outlined regions along the edges and vertices highlight abundant CN and interfaces specifically for CR patients, and **C**) shows these regions for the PD group. (**B**) SCM of combinations comprising more than 0.001% of total cells. Black rectangles indicate SCs with the highest frequency of Tumour, isPLA^−^, Tumour & immune, isPLA^+/−^, and Tumour and immune, isPLA^+^ neighbourhoods for CR patients, and **D**) shows these neighbourhoods for the PD group

We plotted the CN compositions of all samples per each response group onto a barycentric coordinate system. This system demonstrates the scattering of cells, highlighting which SCs are formed by CNs at each vertex. Cells in the proximity of a vertex imply that its neighbourhood predominantly consists of cells from the corresponding CN represented by that vertex. A higher density near a vertex indicates the existence of a spatial context made up of cells “compartmentalised” within that CN. Cells adjacent to the triangle’s borders signify neighbourhoods influenced mainly by two connected CNs, whilst cells situated at the centre comprise neighbourhoods comprising all three CNs. In our data, the density of tumour & immune isPLA^+^ and tumour and immune isPLA^+/−^ in PD patients are markedly higher around the corresponding vertexes (Fig. 6A) compared to the CR group (Fig. 6C).

We identified different combinations of cell neighbourhoods formed by isPLA^+^ tumour cells and various immune cells using SC maps (SCM). Comparison of the SCMs of CR and PD, which represent opposing ends of the response spectrum, indicates that the combination of tumour and immune isPLA^+/−^ with Tumour isPLA^−^ is more prevalent in PD than in CR (Black rectangles). A comparable trend is evident in triple SCMs, where combinations of tumour & immune isPLA^+/−^, tumour PLA^−^, and tumour & immune PLA^+^ are more frequent in PD patients compared to CR, suggesting an increased level of PD-1/PD-L1 interactions in non-responsive patients (Fig. 6B, D).

## Discussion

Over the last decade, ICI has completely revolutionised the way that HNSCC is managed and is one of the most promising cancer treatment approaches. Despite significant efforts to develop innovative anti-PD-1/PD-L1-based immunotherapy techniques aimed at improving clinical responses and reducing immune-mediated toxicity consequences, fewer than 30% of patients respond well to the therapy [40–42]. The current reliance on PD-1 or PD-L1 staining for immunotherapy eligibility is limited, providing little insight into dynamic PD-1/PD-L1 interactions [43]. In this study, we were the first to apply the Navinci in situ proximity ligation assay on HNSCC tissue sections. We could detect PD-1/PD-L1 interactions within the TME using isPLA, which identifies functional PD-1 and PD-L1 actively participating in interactions that can transmit signals, rather than merely detecting standalone markers [44–46]. IsPLA positivity in CNs highlights the abundance of isPLA^+^ macrophage & CD3 T cell-enriched neighbourhoods around the boundary of tumours where the tumour and immune cells reach out. This may represent extensive PD-1/PD-L1 ligation across the tumour edges, specifically in PD patients. However, insufficient markers prevent us from classifying these cells into specific subclasses like M2 macrophages or CD4^+^ T cells. Tumour-associated macrophages (TAMs) are the most common and plastic immune cells within the tumour microenvironment. They are classified into M1 (anti-tumour macrophages) and M2 (pro tumour macrophages) [47–49]. TAMs affect the therapeutic effects of PD-1/PD-L1 inhibitors by regulating the PD-L1 expression in tumour cells, secreting a variety of cytokines to create a tumour-supporting TME and neutralise anti-PD-1/PD-L1 through their Fc-ɣ receptors [50–55]. Furthermore, they can directly express PD-L1 under the TME influence and suppress or apoptosis CD8^+^ T cells, which can affect the efficacy of PD-1/PD-L1 immune checkpoint inhibitors [56].

Similar to our SCMs result, Ji et al. identified spatial barriers made by TAMs around tumours, inhibiting immune cell access and fostering an immunosuppressive microenvironment [57]. In models of non-small cell lung cancer, gastric cancer and pancreatic cancer, TAMs, predominantly M2 TAMs, were consistently found in closer proximity to tumour cells [58–60]. A metastatic melanoma model demonstrated that cytotoxic T cells located at the tumour periphery and close to macrophages showed significant exhaustion. Additionally, macrophages situated near the tumour edge and adjacent to cytotoxic T cells displayed elevated levels of PD-L1 expression [61].

The highest exhaustion of cytotoxic T cells is observed at the macrophage barrier that facilitates increased contact with T cells within the defined “effective interaction distance”, which is typically a radius of less than 20 μm [57]. The proximity of M2 TAMs to tumour cells has also been shown to correlate significantly with patient survival rates and tumour progression [58, 60].

Applying CSF-1R inhibitors like emactuzumab in depleting TAMs which may leads to reduction in macrophage barriers, have shown promising results in reprogramming the TME to favor immune activation [62]. Similarly, CD47-SIRPα axis blockade has shown increasing macrophage-mediated phagocytosis of tumour cells, which can complement existing therapies. These approaches highlight the potential for targeting macrophage barriers to improve therapeutic outcomes in patients with resistant tumours [63].

Our study similarly observed a dense aggregation of macrophages and tumour neighbourhoods around the tumour-stroma boundary in non-responsive patients rather than responders.

A high intratumoral Treg density has been demonstrated to create an expansive immunosuppressive milieu, facilitating tumour cell evasion from immune surveillance [64, 65]. It has been shown that the presence of Treg cells close to the tumour margins enables tumour cells to escape the immune response, whereas the deficiency of Treg cells in the pan-stroma contributes to heightened inflammation that facilitates tumour invasion [66]. Accordingly, our findings suggest that it is likely that the CD3 T cells we found are regulatory T cells working with TAMs to suppress immune responses. Observing a layer of isPLA^+^ immune & tumour cell neighbourhoods around the boundary of tumour-stroma regions, especially in non-responders, aligns with the current understanding of the role of TAMs and Tregs in immune suppression and tumour progression.

Interestingly, there are roughly similar regions in other response groups as well, but their thickness, coverage and structure are entirely different, limited to tiny spots with scattered isPLA^+^ macrophage-tumour neighbourhoods in the PR group. Moreover, isPLA^+^ B cell and T cell aggregates, resembling tertiary lymphoid structures (TLS), were observed close to the tumour boundaries in CR patients. PD-1/PD-L1 interactions between B cells and T cells within TLSs have been shown to regulate immune response and anti-tumour immune functions [67–70]. The existence of TLSs correlates with enhanced responses to ICI therapy and higher patient survival rates [68–70]. TLSs boost anti-tumour activity by promoting dendritic cell antigen presentation, enhancing B-cell-mediated immunity, and sustaining T-cell activation, persistence, and survival over extended antigen exposure [68]. By enhancing immune surveillance and recruiting diverse immune cell types, TLSs can enhance the efficacy of immunotherapies, rendering them potential biomarkers for predicting treatment responses [71–75].

In this study, we hypothesise that forming macrophage-enriched immune-tumour cell complexes might represent a barrier, limiting the infiltration of immune cells like cytotoxic T cells and contributing to the immune escape mechanism frequently observed in non-responsive HNSCC cases. The density and spatial arrangement of macrophages observed in this study can influence clinical outcomes, as high macrophage infiltration often correlates with poor prognosis and reduced therapy efficacy ​ [76, 77]. On the other hand, in patients who responded to the immunotherapy, we observed a considerable aggregation of B cell neighbourhoods either standing alone by themselves or within TLSs close to the tumour-stroma boundary. Comparing cellular neighbourhoods that consider isPLA positivity with those that do not, demonstrate the clear advantage of in situ proximity ligation technology in revealing distinct differences in immune-tumour cell interactions and cell architecture within the tumour microenvironment between responders and non-responders.

The application of isPLA assay for the first time in HNSCC indicates that macrophages, T cells and B cells located in various spatial analyses potentially influence response to immunotherapy. The findings suggest that macrophage-tumour aggregation in the peripheral stroma of tumours may significantly contribute to malignant progression while B cell aggregates can enhance the likelihood of treatment response.

isPLA technology allows us to visualize the interactions between PD-1/PD-L1 through positive isPLA signals rather than relying on the colocalisation of single proteins within the tumour.

The study’s limitations include a limited patient cohort size and variable patient numbers across response groups. Furthermore, antibody availability and staining efficacy limitations restricted our ability to visualise specific cell types. Consequently, our data provides only a limited view of the wider immune landscape. The application of manual thresholding to classify the signal intensity into high and low lacked a standardised method for signal spectrum identification. It depended on the quality and intensity of each tissue sample. Regardless of the inclusion or exclusion of isPLA, the identified spatial neighbourhoods are applicable only to this tumour cohort. Other datasets may define these neighbourhoods differently, meaning our findings may not fully apply to broader contexts. The absence of a standardised approach for scoring PD-1/PD-L1 interactions poses a methodological challenge. The differential expression of PD-1 and PD-L1 across various cancer types and stages complicates the interpretation of their interactions. Lastly, we excluded the stable disease (SD) group from our analysis due to inconsistent and highly variable responses within this group that might obscure clear conclusions about treatment efficacy.

## Conclusion

Our study, which is the first to investigate the function of PD-1/PD-L1 interactions in the immunotherapy response of HNSCC patients, has uncovered a crucial link between these interactions and the spatial arrangement of immune cells and tumours. This complex interplay is essential for understanding the mechanisms behind the varying responses to immune checkpoint inhibitors in HNSCC patients. While more research and different approaches are required for validation, our results provide a novel observation: unique configurations of isPLA^+^ macrophages, T cells and B cells interacting with immune cells at the tumour periphery correlate with the response to ICI therapy. The findings align with current immunological concepts and emphasise the need to directly study biomarker interactions rather than focus on them separately to improve treatment outcomes. These methodologies offer a potent platform for enhancing treatment strategies and advancing diagnostic pathologies providing a foundation for stratifying patients for immune checkpoint inhibitor (ICI) therapies based on the spatial dynamics of PD-1/PD-L1 interactions within the tumor microenvironment. They also open new avenues for research focused on overcoming immune evasion in non-responsive patients.

## Electronic supplementary material

Below is the link to the electronic supplementary material.

Supplementary Material 1

## Data Availability

The data associated with this manuscript will be made available by reasonable request with the corresponding author.
