# Supplementary material for: Spatial interaction mapping of PD-1/PD-L1 in head and neck cancer reveals the role of macrophage-tumour barriers associated with immunotherapy response
Source: J Transl Med. 2025 Feb 12;23:177. doi: 10.1186/s12967-025-06186-y (PMC11818323; doi:10.1186/s12967-025-06186-y)
Supplement: Supplementary file 1 — Supplementary Material 1 [file 12967_2025_6186_MOESM1_ESM.docx]

**
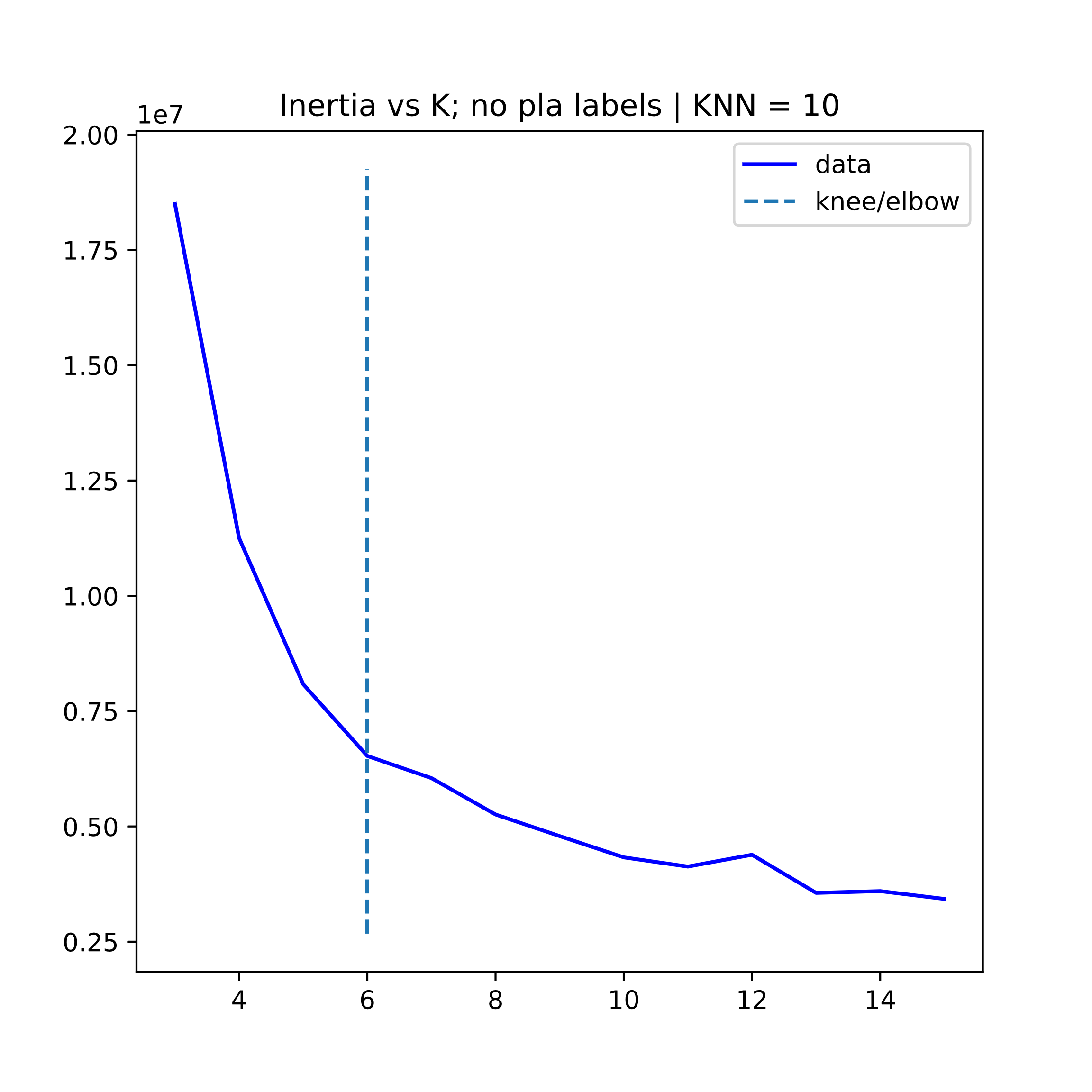

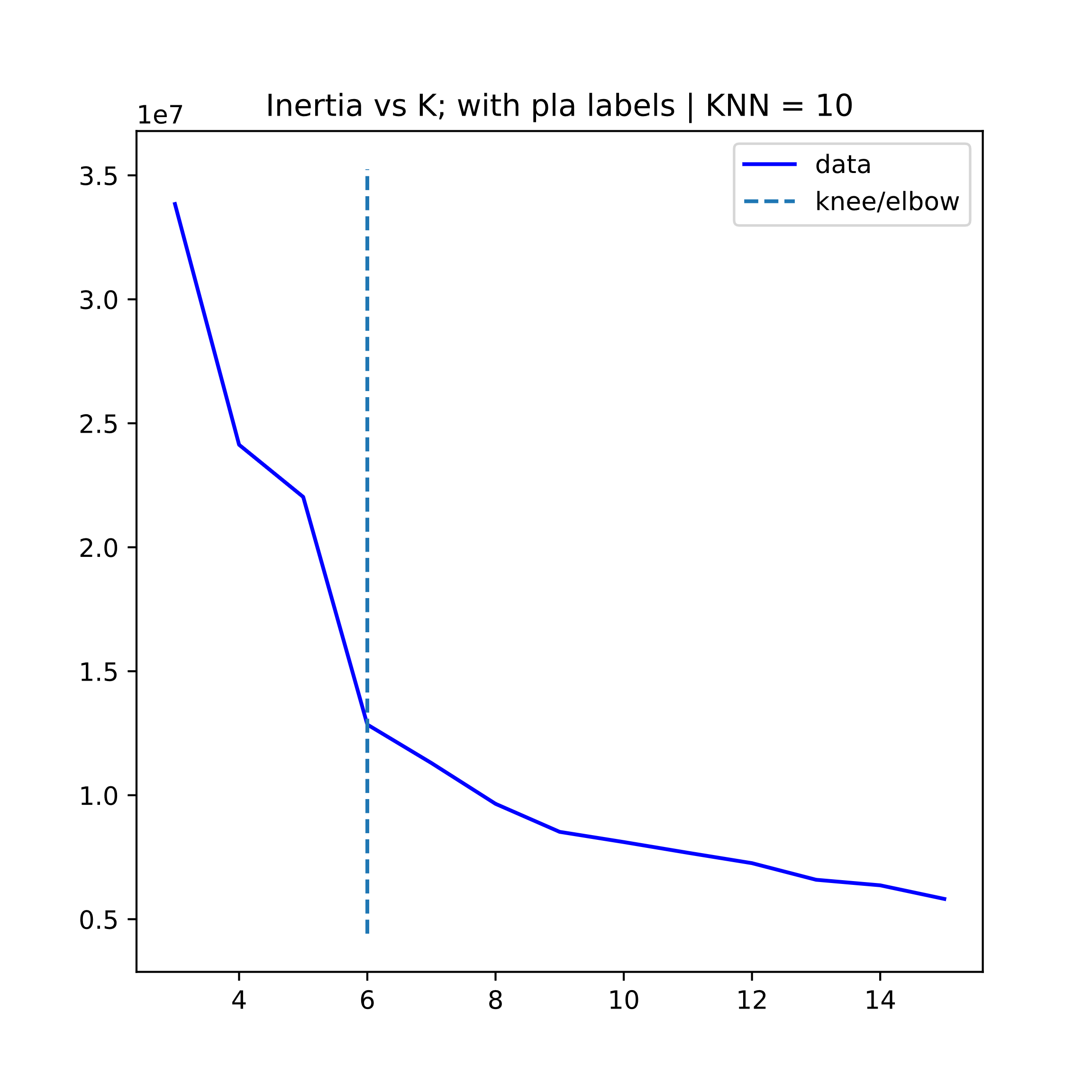
Supplementary figures:**

K

Inertia

K

Inertia

**B**

**A**

**Figure 1:** A feature matrix was generated using the 10 nearest neighboring cells to identify clusters with similar characteristics. KMeans clustering was performed on this matrix, testing different cluster numbers (K) from 3 to 15. For each K, the sum of squared distances (inertia) was measured to assess the compactness of the clusters. The optimal number of clusters was selected by finding the elbow point in the plot of inertia versus K in the CNs with PLA labels (A) and without PLA labels (B), with the Kneedle algorithm used to pinpoint this elbow systematically. The dashed blue line is the ‘elbow’ identified by the Kneedle algorithm.


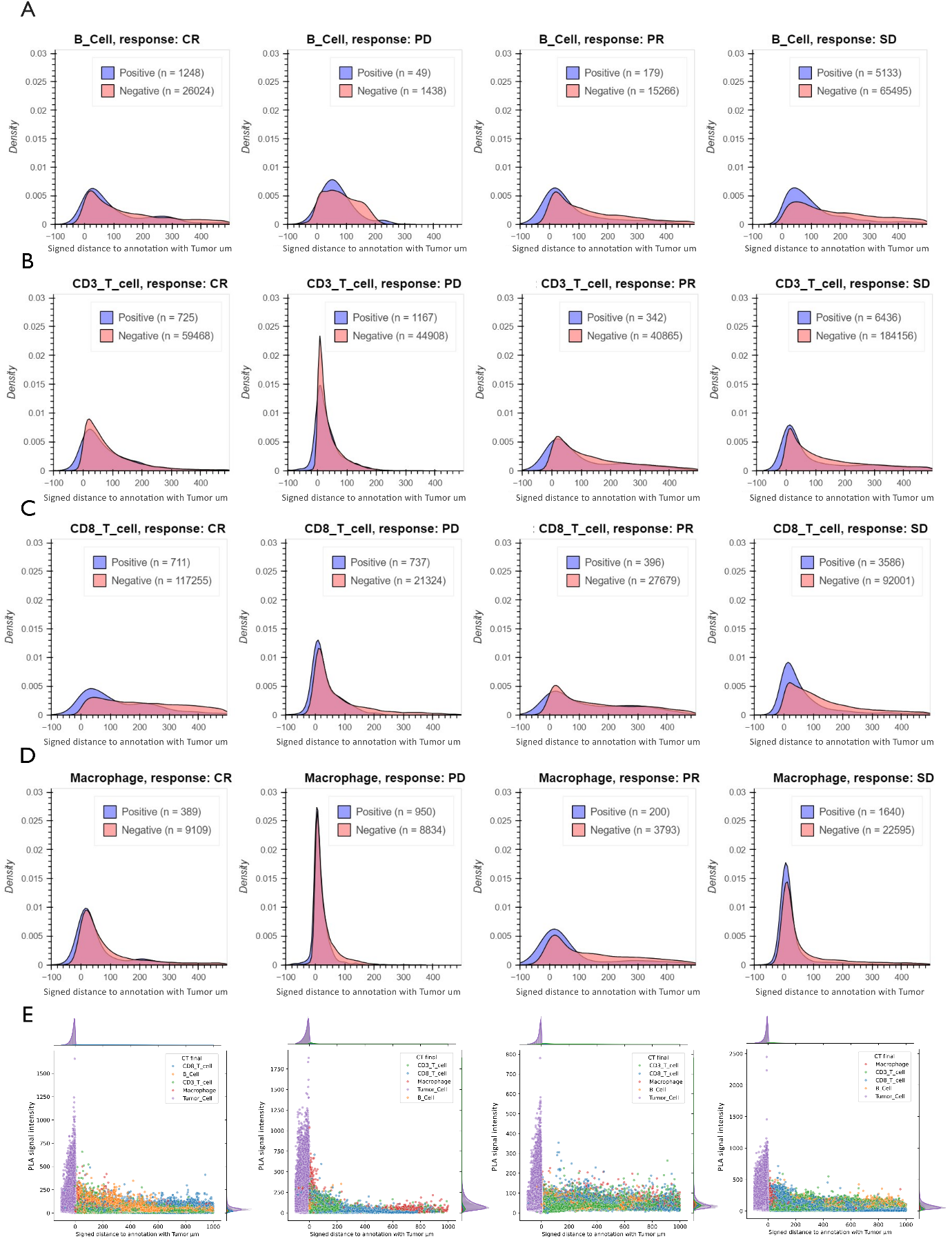


Figure 2: Density plots showing the distance to the tumour boundary, normalized cell count, and isPLA status for B cells (panel A), CD3 T cells (panel B), CD8 T cells (panel C) and macrophages (panel D) across response groups.
